# Screen Exposure and Early Childhood Development in Resource-Limited Regions: Findings From a Population-Based Survey Study

**DOI:** 10.2196/68009

**Published:** 2025-05-15

**Authors:** Yuyin Xiao, Dorien Emmers, Shanshan Li, Hanwen Zhang, Andrew Rule, Scott Rozelle

**Affiliations:** 1 Stanford Center on China’s Economy and Institutions, Stanford University Stanford, CA United States; 2 Center for Innovation in Global Health (CIGH), Stanford University Stanford, CA United States; 3 Chinese Studies Group, Katholieke Universiteit Leuven (KU Leuven) Leuven Belgium; 4 Department of Economics, Katholieke Universiteit Leuven (KU Leuven) Leuven Belgium; 5 Center for International Cooperation and Disciplinary Innovation of Income Distribution and Public Finance (111 Center) Zhongnan University of Economics and Law Wuhan, Hubei China

**Keywords:** screen time, digital media viewing, screen use, infancy, rural area, low- and middle-income countries

## Abstract

**Background:**

The content of children’s screen exposure and interactive coviewing with caregivers are important determinants of early childhood development (ECD) that have been overlooked in past research in resource-limited rural regions. Given the prevalence of digital devices and diverse digital content today, determining screen use practices that minimize the negative impacts on children’s development is crucial for promoting healthy screen use among children.

**Objective:**

This study aims to examine screen exposure among children aged <3 years in rural China and investigate its relationship with ECD outcomes, focusing on duration, content, coviewing, and interaction.

**Methods:**

The sample includes all children aged between 6 and 26 months and their primary caregivers residing in the study area. A survey of screen exposure and household characteristics was conducted for 1052 eligible households. Caregivers reported the duration of screen exposure, defined as the average daily screen time over the past month; the content of exposure, defined by the time spent on educational and child-friendly content; and the caregiver’s presence and interaction with the child during exposure. ECD outcomes were evaluated using the third edition of the Bayley Scales of Infant and Toddler Development assessment scale and the Brief Infant Toddler Social Emotional Assessment. Ordinary least squares regression, logistic regression, and chi-square tests were conducted.

**Results:**

In total, 28.23% (297/1052) of the children in our sample were first exposed to screens before the age of 12 months. Children exposed to screens had an average daily screen time of 27.57 (SD 38.90) minutes. Children who were exposed to screens before the age of 12 months and those who had longer screen time between the ages of 12 and 18 months were more likely to be at risk of motor developmental delays. Children exposed to educational content for >15 minutes on a daily basis had fewer social-emotional or behavioral problems than those with no screen exposure and a lower risk of delay in motor skills development than those exposed to educational content for <15 minutes on a daily basis. Caregiver interaction during screen exposure was associated with a lower risk of cognitive and language delays and better socioemotional skills.

**Conclusions:**

The type of content viewed and how caregivers engaged in children’s screen time were strongly associated with ECD outcomes. Guiding parents to select educational content for their children and engaging in interactive coviewing may better protect children from the negative effects of screen exposure. The findings complement conclusions regarding the impact of screen exposure on ECD in resource-limited rural areas.

## Introduction

### Background

Dramatic increases in electronic device use and exposure among young children in recent years have spurred efforts to determine the impact of screen exposure on early childhood development (ECD) outcomes. The first 3 years of life are a crucial period for development, and development during this stage is closely associated with academic performance and employment later in life [1]. Many researchers have suggested that screen exposure during early childhood has negative effects on attention [2], behavior [3,4], and academic performance [5]. The effects increase with longer screen time and earlier initial exposure, regardless of the type of electronic content. Consequently, the American Academy of Pediatrics has long advised that screen time be held to a minimum during the first few years of life, with its most recent guidelines recommending that children aged <18 months avoid screen exposure entirely [6]. However, other studies have suggested that certain types of screen exposure may be less harmful to young children and even promote cognitive development. For instance, there is evidence that child-friendly educational programs can have a positive impact on learning and education outcomes [7,8]. Virtual characters in videos have also been found to foster the formation of social relationships and support socioemotional development [9].

Determining how content type, coviewing, and interaction with caregivers affect the impact of screen exposure on ECD remains a key priority for researchers, especially in resource-limited rural areas where rates of early screen exposure are increasingly high, but evidence of its effect on ECD remains scarce. One systematic review found that children aged <5 years in low- and middle-income countries spent between 0.1 and 5 hours per day using screens [10]. Another study found that the average daily screen time of children aged between 0 and 2 years in middle-income countries exceeded 1 hour [11]. The negative consequences of excessive screen exposure for ECD have also been well documented in resource-limited settings. A study of children aged between 0 and 5 years in Brazil found that each additional hour of screen time was associated with increased risks of developmental delay in communication, problem-solving, and personal-social skills [12]. Similarly, a small-sample cohort study found that excessive screen time in early childhood was associated with poor cognitive and socioemotional development [13]. However, these studies failed to account for variations in content type, whether caregivers engaged in coviewing, and whether they interacted with their children during coviewing. A study that assesses the duration and content of child screen exposure, caregiver coviewing, and child-caregiver interaction during coviewing in resource-limited rural areas will help identify screen use practices that minimize the negative impacts on ECD. This insight can inform future research and policy efforts aimed at encouraging healthy screen use practices among caregivers and supporting positive ECD in their children.

Researchers have measured the content children are exposed to on screens in terms of its educational value, interactivity, age-appropriateness, and more [14]. Studies conducted in English-speaking, high-income societies have found that, under similar exposure durations, educational television programs such as Blue’s Clues and Sesame Street enhanced children’s cognitive development, while commercial programs had no significant effect [8,15]. However, no similar study has been replicated among children in resource-limited areas, who are exposed to vastly different cultural environments and educational programs compared to their peers in English-speaking, high-income societies. Most previous studies in resource-limited regions have focused on the effects of either the duration or the content of screen exposure on ECD, whereas few have considered duration and quality in junction [13,16]. In contrast, coviewing refers to whether children are exposed to screens alone or in the presence of others [17]. A small-sample study showed that interactive coviewing with caregivers can enhance children’s attention and ability to learn new vocabulary more effectively than solitary viewing [18], especially when children are younger [19]. Given the prevalence of screens and diversity of digital content for children today, identifying types of content and coviewing modalities that promote healthy development is essential for maximizing the positive effects of screen exposure on ECD while minimizing its risks.

Another group of often overlooked factors of early screen exposure and ECD are household characteristics that predict frequent screen exposure or exacerbate its negative developmental consequences. For example, previous studies have suggested that migrant children in rural China, who are often raised by grandparents because their parents migrated to urban areas for employment, tend to have significantly higher screen exposure durations [20]. Children from families with lower socioeconomic status have also been found to spend more time engaging in screen-based activities such as watching television [21,22]. Because many caregivers in low-income areas have limited time and resources to engage in interactive activities with their children, these children are more likely to have prolonged periods of unsupervised screen exposure and be affected by its negative effects. Therefore, identifying children and households at the highest risk of negative developmental impacts from screen exposure is crucial for future research and policy interventions.

Finally, there is a pressing need for research on the prevalence and quality of early screen exposure among children aged <3 years in rural China. China’s rural communities remain significantly economically underdeveloped compared to the country’s urban areas [23], and the rate of developmental delays among rural children remains high [24]. In contrast, electronic device use has soared in rural China in the past decade. Results from the China Family Panel Studies, a national representative survey, show that the proportion of China’s rural population that used mobile phones increased from 68.7% in 2010 to 98.9% in 2018 [25]. Despite persisting developmental delays and widespread screen use, there is still a lack of evidence on screen exposure among children in rural China and its effects on their development. Specifically, there is almost no empirical research on screen exposure among children aged <3 years in rural areas of China. By examining the status quo of early screen exposure in rural China and its development impacts, we aim to gain insights that can guide future interventions to promote healthy screen use and ECD in China and in other resource-limited countries.

### Objectives

The overall goal of this study is to examine screen exposure and its associations with ECD outcomes in rural China. To reach this goal, we answered the following questions: (1) How prevalent is screen exposure among children aged <3 years in rural China? (2) Is early or excessive screen exposure associated with cognitive, language, motor, and social-emotional development? (3) Do these associations differ based on the type of screen content and whether caregivers participated in interactive coviewing? (4) What characteristics do households where children have excessive screen exposure have in common?

## Methods

### Design and Participants

The data in this study were collected from 6 counties in Sichuan Province, a southwestern province of China, between May and October 2023. In 2022, it ranked in the bottom third in disposable income per capita among all provinces in China. After randomly selecting 8 counties, we excluded 2 in mountainous areas with low population density. In the remaining 6 counties, villages that met all the following requirements were included: (1) the majority of the village population was of Han ethnicity, the largest ethnic group in China, and this was done to limit the effect of language barriers faced by some non-Han ethnic groups on data collection; (2) the village had a population of at least 1000, considering that villages with smaller populations typically have very few children aged <3 years; and (3) the village had at least 1 local partner to assist in data collection, typically a representative from the local branch of the Women’s Federation. A total of 125 rural villages met all the criteria. In total, 1052 eligible households residing in these villages with children within our target age range (6-26 months) were recruited to participate in the study. Enumerators who had undergone standardized training administered a survey to the children’s primary caregivers, who were identified by each household as the individual most responsible for the child’s care and were typically the child’s mother or grandmother.

### Measures

In the survey administered to caregivers, we collected three categories of data: (1) the duration, content, and coviewing of children’s screen exposure; (2) ECD outcomes; and (3) demographic characteristics of the children and their households.

#### Duration and Content of Screen Exposure and Coviewing During Exposure

The duration of a child’s screen exposure was measured by the average time (in hours) the child spent on screens, including television, computers, tablets, mobile phones, and other devices, per day in the past month as reported by the child’s primary caregiver. In addition, the caregiver reported the age (in months) at which the child was first exposed to electronic screens.

The content of a child’s screen exposure was measured using two indicators: (1) the daily duration of exposure to educational content (in hours) and (2) the proportion of daily screen time spent on child-friendly content in the past month. These 2 indicators were calculated based on caregiver responses to the following questionnaire items: (1) “In the past month, on average, how many hours per day did your child spend on educational screen content?” and (2) “In the past month, how many hours per day did your child spend on child-friendly screen content?” as well as “How many hours per day did your child spend on digital screens (including television, computers, tablets, mobile phones, etc)?” Both indicators were based on caregiver reports and definitions. While caregivers’ definitions for educational and child-friendly content may vary, the measure reflects the extent to which caregivers intentionally monitor and select digital content for their child.

The coviewing of a child’s screen exposure was measured by the frequency of caregiver company and interaction with the child during their screen exposure. Caregivers reported how often family members were present during the child’s screen time (half or less than half of the time vs more than half of the time) and how frequently they interacted or communicated with the child during screen exposure in the past month.

#### ECD Outcomes

The ECD outcomes of the sample were measured using the third edition of the Bayley Scales of Infant and Toddler Development (Bayley-III) [26] and its simplified version (refer to Multimedia Appendix 1 for a detailed description). Bayley-III assesses cognitive skills, expressive and receptive language, and gross and fine motor skills through observational evaluation. Children were given tasks of varying difficulty levels and received a binary score of 1 for every completed task and 0 for every failed task. The test stops when a child fails 5 consecutive tasks. Assessors who participated in a 1-week practice training before data collection administered the assessment using a standardized set of toy kits and detailed record sheets.

We also assessed children’s social-emotional development using the Brief Infant Toddler Social Emotional Assessment (BITSEA), which measures competence delays and social-emotional or behavioral problems. The primary caregiver was asked to report whether the child had exhibited a series of behaviors and the frequency of their occurrence and assign corresponding scores (0=not true or rarely, 1=somewhat true or sometimes, and 2=very true or often). Scores for each subdomain were calculated by summing the total scores. Higher scores in either the competence subdomain or the social-emotional or behavioral problems subdomain correspond to a higher risk of developmental delay in that subdomain. The BITSEA has been validated as an effective tool for behavioral problems and developmental competence levels globally [27]. Internal consistency was 0.66 for the BITSEA Competence scale and 0.81 for the BITSEA Problem scale. Construct validity indicated that the factor structure of the BITSEA scales was adequate, with each item being well associated with its corresponding original scale. The composite reliability was 0.68 for the BITSEA Competence scale and 0.82 for the BITSEA social-emotional or behavioral Problem scale. Both model fit indices reached acceptable levels, with root mean square error of approximation=0.06. Because the BITSEA is only validated for children aged between 12 and 36 months, we only administered it to children aged >12 months in this study.

#### Demographic Information

We collected information on the child’s sex (female or male), age (aged <12 or ≥12 months, <18 or ≥18 months, and <26 months), whether they had siblings (yes or no), their primary caregiver’s age (aged <25 or ≥25 years, <30 or ≥30 years, <40 or ≥40 years, and <50 or ≥50 years), level of education (junior high school or below or high school or above), relationship with the child (mother, father, grandparent, or others), mental health status, and family wealth. Caregiver mental health was measured using the Depression Anxiety Stress Scale, which evaluates depression, anxiety, and stress [28]. The scale has been shown to perform with reasonable reliability, with Cronbach α coefficients ranging from 0.81 to 0.85. The scale also has good construct validity, with the factor loadings of the measured items being >0.500. The model fit indices reached acceptable levels, with root mean square error of approximation=0.07. Higher scores indicate a higher risk of mental health issues. Household wealth was estimated based on the assets owned by the family. Caregivers were asked to report ownership of the following items: household appliances (eg, toilets, water heaters, washing machines, refrigerators, and air conditioners), electronic devices (eg, televisions, computers, and tablets), transportation (eg, motorcycles, electric bikes, and cars), and real estate. We calculated a household wealth index using a categorical principal component analysis approach for variables of mixed measurement levels (nominal or ordinal) that may not be linearly related to each other. Then, we standardized the index to transform it into a value ranging from 0 to 1, where a larger value indicates more household wealth.

### Statistical Analysis

Our data analysis is structured as follows. We first described average daily screen exposure duration and age of first exposure among children aged <3 years in rural China. Then, we tested the relationship between the duration of screen exposure and ECD outcomes in different age groups by ordinary least squares (OLS) regression and logistic regression. OLS regression was used for continuous ECD outcome variables, while logistic regression was applied for binary ECD outcomes. Before modeling, we checked key assumptions and variance inflation factors for each regression. For OLS models, we used diagnostic plots to assess linearity, homoscedasticity, and normality of residuals. For logistic models, we assessed model fit using the Hosmer-Lemeshow test. Similarly, we tested whether the content and coviewing of screen exposure impacted ECD outcomes by estimating the OLS regression and logistic regression. Multimedia Appendix 1 shows the specific statistical formulas and parameter settings. Finally, we conducted a chi-square test analysis to explore the individual characteristics of children aged <3 years who are more prone to excessive screen exposure. All statistical analyses were performed using R (version 4.1.0; R Foundation for Statistical Computing).

### Ethics Approval

This study has been approved by the institutional review board of Stanford University (protocol #63680). Informed consent was obtained from all participants, who were also informed of their right to withdraw from the study at any time. All data were anonymized and stripped of identifying information. Participants were offered a gift valued at approximately US $5 upon completion of the questionnaire.

## Results

### Demographic Characteristics

The demographic characteristics of all children and their primary caregivers in the sample are shown in Table 1. Of the 1052 children, 561 (53.33%) were male, 386 (36.69%) were aged between 18 and 26 months at the time of survey administration, and 469 (44.58%) were firstborn. Of the 1052 primary caregivers, 908 (87.06%) were female, 731 (69.49%) were mothers, 92 (8.75%) were fathers, and 221 (21.01%) were grandparents. Most caregivers (354/1052, 33.65%) were aged between 25 and 30 years or between 30 and 40 years (300/1052, 28.52%), and 13.02% (137/1052) of the caregivers were aged <25 years. Finally, 60.27% (634/1052) of the caregivers only completed junior high school education or less. Because some samples only completed a portion of the ECD outcome evaluations, Table S1 in Multimedia Appendix 1 presents the demographic characteristics of the subsamples, with 818 samples completing the Bayley-III assessment, 937 samples completing the BITSEA social-emotional problems assessment, and 960 samples completing the BITSEA competence delays assessment.

**Table 1:** Characteristics of children and their primary caregivers (N=1052).

Variables			Participants, n (%)
**Child’s sex**			
	Female	491 (46.67)	
	Male	561 (53.33)	
**Child’s age (mo)**			
	<12	315 (29.94)	
	≥12 to <18	351 (33.37)	
	≥18 to <26	386 (36.69)	
**Firstborn**			
	No	583 (55.42)	
	Yes	469 (44.58)	
**Caregiver’s relationship**			
	Mother	731 (69.49)	
	Father	92 (8.75)	
	Grandparent	221 (21.01)	
	Others	8 (0.76)	
**Caregiver’s sex**			
	Female	908 (87.06)	
	Male	135 (12.94)	
**Caregiver’s age (y)**			
	<25	137 (13.02)	
	≥25 to <30	354 (33.65)	
	≥30 to <40	300 (28.52)	
	≥40 to <50	83 (7.89)	
	≥50	170 (16.16)	
**Caregiver’s education**			
	Junior high school or below	634 (60.27)	
	High school or above	407 (38.69)	

### Duration of Screen Exposure

Table 2 describes the age of first screen exposure and the duration of daily screen exposure among children in our sample. Of the 1052 children, 432 (41.06%) had never been exposed to screens, 73 (6.94%) were first exposed between 18 and 26 months, 250 (23.76%) were first exposed between 12 and 18 months, and 297 (28.23%) were first exposed before 12 months of age. In the month before data collection, the mean daily screen time of all children was 16.25 (SD 32.79) minutes, or 27.57 (SD 38.90) minutes when excluding children with no screen exposure; 45.91% (483/1052) of the children had 0 hours of screen time daily that month, 43.82% (461/1052) had <1 hour, and 10.27% (108/1052) had ≥1 hour. Table S2 in Multimedia Appendix 1 shows the average screen time and age of first exposure for subsets of our sample that completed each of the assessments.

**Table 2:** Summary statistics of screen exposure quantity (N=1052).

Variables			Values
**Age of first screen exposure (mo), n (%)**			
	No exposure	432 (41.06)	
	≥18 to <26	73 (6.94)	
	≥12 to <18	250 (23.76)	
	<12	297 (28.23)	
**Screen exposure duration (h), n (%)**			
	No exposure	432 (41.06)	
	0	51 (4.85)	
	<1	461 (43.82)	
	≥1	108 (10.27)	
**Screen exposure duration (min), mean (SD)**			
	Entire sample	16.25 (32.79)	
	Children with exposure	27.57 (38.90)	

### Screen Exposure Duration and Developmental Delay

We first tested the relationship between age of initial screen exposure and developmental delay. The results are shown in Table 3. Overall, compared to children with late or no screen exposure, children exposed to screens before 12 months of age were significantly more likely to have developmental delays in motor skills (*P*<.001) but showed no significant differences in cognitive (*P*=.54) and language development (*P*=.51).

**Table 3:** Relationship between time of initial exposure and developmental delay (N=818).

Variables		Age of first screen exposure (mo)					*P* value
		No exposure, n (%)	18-26, n (%)	12-18, n (%)	<12, n (%)		
**Cognition**							.54
	Normal	209 (61.47)	27 (50.94)	114 (60.64)	139 (60.70)		
	Delay	131 (38.53)	26 (49.06)	74 (39.36)	90 (39.30)		
**Language**							.51
	Normal	223 (66.97)	37 (71.15)	120 (64.86)	140 (61.95)		
	Delay	110 (33.03)	15 (28.85)	65 (35.14)	86 (38.05)		
**Motor**							<.001
	Normal	244 (73.27)	45 (86.54)	168 (90.81)	162 (71.68)		
	Delay	89 (26.73)	7 (13.46)	17 (9.19)	64 (28.32)		

We then identified periods of early childhood in which the associations between screen exposure and developmental delays in cognitive, language, and motor skills were the strongest. Table 4 and Tables S1 and S2 in Multimedia Appendix 2 present the associations between age of first screen exposure, daily screen time, and ECD outcomes for children in different age groups. We found no significant association between the age of first screen exposure and developmental delay in any of the age groups. In addition, there were no significant associations between daily screen time and developmental delay among children aged <12 months (the youngest age group) or those aged between 18 and 26 months (the oldest age group). However, increased screen exposure is associated with a higher risk of motor developmental delay among children aged between 12 and 18 months (*P=*.01).

**Table 4:** Screen exposure duration and child development (subgroup age <12 mo; N=250).

	Age of first screen exposure <12 mo, odds ratio^a^ (95% CI)	Daily screen time, odds ratio^a^ (95% CI)
Delay in cognition	0.994 (0.530-1.862)	1.000 (0.985-1.022)
Delay in language	1.054 (0.532-2.086)	1.006 (0.986-1.026)
Delay in motor skills	1.651 (0.870-3.132)	0.987 (0.967-1.007)

^a^The no exposure group used as reference. Control variables included baby’s age, baby’s sex, firstborn or not, household asset index, type of caregiver, caregiver’s sex, caregiver’s age, and caregiver’s education.

We further tested the association between screen exposure and social-emotional problems among children aged >12 months who completed the BITSEA scale. We found no significant association between age of first screen exposure and social-emotional or behavioral outcomes among either children aged between 12 and 18 months or between 18 and 26 months. Similarly, there was no significant association between daily screen time and social-emotional or behavioral outcomes in either age group.

### Screen Exposure Content, Coviewing, and Developmental Delay

Next, we explored the relationship between content of screen exposure and ECD outcomes. The results are shown in Tables 5 and 6. The proportion of child-friendly content consumed was not significantly associated with the risk of cognitive, language, or motor delays. Children exposed to educational content for <15 minutes on a daily basis experienced more social-emotional or behavioral problems than those with no screen exposure (*P*<.01). They also showed significantly higher rates of delay in motor skills development than those exposed to educational content for ≥15 minutes on a daily basis (*P*=.03). However, among children with screen exposure, neither screen time spent on educational content nor the proportion of child-friendly content to total screen time was significantly associated with social-emotional or behavioral problems.

**Table 5:** Content of screen exposure and child development (cognition, language, and motor skills).

	Most viewed content				Time spent on educational content (min)		
	Non–child-friendly content, odds ratio^a^ (95% CI)	Child-friendly content, odds ratio^a^ (95% CI)	*P* value^b^	<15 min, odds ratio^a^ (95% CI)		≥15 min, odds ratio^a^ (95% CI)	*P* value^b^
Delay in cognition (n=818)	1.065 (0.535-2.118)	1.024 (0.730-1.438)	.62	0.113 (0.814-1.567)		0.759 (0.437-1.321)	.09
Delay in language (n=818)	0.638 (0.295-1.384)	1.181 (0.830-1.679)	.91	1.153 (0.821-1.619)		1.112 (0.634-1.950)	.29
Delay in motor skills (n=818)	1.241 (0.565-2.727)	0.949 (0.654-1.486)	.68	1.104 (0.749-1.629)		0.893 (0.412-1.932)	.03

^a^No exposure group used as reference. Control variables included baby’s age, baby’s sex, firstborn or not, household asset index, type of caregiver, caregiver’s sex, caregiver’s age, and caregiver’s education.

^b^Comparison between children with exposure to more versus less child-friendly or educational content.

**Table 6:** Content of screen exposure and child development (social- emotional behavior).

	Most viewed content				Time spent on educational content (min)		
	Non–child-friendly content, estimate^a^ (SE)	Child-friendly content, estimate^a^ (SE)	*P* value^b^	<15 min, estimate^a^ (SE)		≥15 min, estimate^a^ (SE)	*P* value^b^
Social-emotional behavior problems (n=937)	1.233 (0.933)	0.690 (0.440)	.94	1.046^c^ (0.426)		−0.244 (0.690)	.09
Social-emotional behavior competencies (n=960)	0.502 (0.442)	0.191 (0.217)	.78	0.237 (0.211)		0.351 (0.345)	.35

^a^No exposure group used as reference. Control variables included baby’s age, baby’s sex, firstborn or not, household asset index, type of caregiver, caregiver’s sex, caregiver’s age, and caregiver’s education.

^b^Comparison between children with exposure to more versus less child-friendly or educational content.

^c^*P*<.01.

We further tested the relationship between the coviewing of screen exposure and developmental delays. Tables 7 and 8 show the associations between caregiver company and interaction during screen exposure and ECD outcomes. The proportion of screen time that children spent in the company of others was not significantly associated with cognitive, language, or motor developmental delays. In contrast, children who spent more than half of their screen time interacting with their caregivers had significantly lower rates of cognitive and language developmental delays than those who spent less than half of their screen time interacting with caregivers (*P*=.03), although we found no significant association between proportion of screen time spent interacting with caregivers and motor developmental delay. In addition, children who spent more than half of their screen time interacting with caregivers also showed higher social-emotional competency than children with no exposure (*P*=.01). Overall, children who experienced more interaction in their screen time showed higher social-emotional competence and fewer delays in cognitive and language development.

**Table 7:** Coviewing of screen exposure and child development.

	Proportion of screen time spent with caregiver present				Proportion of screen time spent interacting with caregivers		
	Less than half, odds ratio^a^ (95% CI)	More than half, odds ratio^a^ (95% CI)	*P* value^b^	Less than half, odds ratio^a^ (95% CI)		More than half, odds ratio^a^ (95% CI)	*P* value^b^
Delay in cognition (n=818)	1.333 (0.895-1.984)	0.853 (0.593-1.227)	.05	1.144 (0.805-1.625)		0.839 (0.552-1.276)	.03
Delay in language (n=818)	1.290 (0.540-1.948)	1.007 (0.691-1.466)	.52	1.338 (0.931-1.922)		0.806 (0.518-1.253)	.03
Delay in motor skills (n=818)	1.308 (0.813-2.105)	0.889 (0.567-1.395)	.80	1.258 (0.829-1.910)		0.736 (0.424-1.276)	.06

^a^No exposure group used as reference. Control variables included baby’s age, baby’ s sex, firstborn or not, household asset index, type of caregiver, caregiver’s sex, caregiver’s age, and caregiver’s education.

^b^Comparison between children with exposure to more versus less screen time with caregiver company or interaction with caregivers.

**Table 8:** Coviewing of screen exposure and child development.

	Proportion of screen time spent with caregiver present				Proportion of screen time spent interacting with caregivers		
	Less than half, estimate^a^ (SE)	More than half, estimate^a^ (SE)	*P* value^b^	Less than half, estimate^a^ (SE)		More than half, estimate^a^ (SE)	*P* value^b^
Social-emotional behavior problems (n=937)	0.906 (0.523)	0.806 (0.468)	.96	0.904^c^ (0.461)		0.676 (0.535)	.79
Social-emotional behavior competencies (n=960)	0.140 (0.258)	0.358 (0.230)	.27	0.032 (0.227)		0.665^d^ (0.261)	.01

^a^No exposure group used as reference. Control variables included baby’s age, baby’ s sex, firstborn or not, household asset index, type of caregiver, caregiver’s sex, caregiver’s age, and caregiver’s education.

^b^Comparison between children with exposure to more versus less screen time with caregiver company or interaction with caregivers.

^c^*P*<.05.

^d^*P*<.01.

### Risk Factors of Excessive Screen Exposure

Finally, we examined whether children with prolonged screen exposure, early exposure, or exposure to less educational content differed in their sociodemographic characteristics compared to other children, as shown in Tables 9 and 10. As shown in Table 9, children whose caregivers experienced stress and anxiety symptoms were more likely to be exposed to screens at an early age; children aged >18 months and whose caregivers experienced depressive symptoms were more likely to have longer daily screen time. As shown in Table 10, among children with screen exposure, children aged <12 months and those with less educated caregivers were more likely to be exposed to educational content for <15 minutes on a daily basis. Less educated caregivers also interacted with their children less during their screen time. Neither the child’s sex, the number of siblings, nor the caregiver’s sex, age, or relationship with the child was significantly associated with the duration, content, or coviewing of screen exposure.

**Table 9:** Differences in sample characteristics between different screen exposure quantity groups (n=1052).

		Daily screen time					Age of first screen exposure					
		<1 h, n (%)	≥1 h, n (%)	*P* value		<12 months, n (%)		≥12 months and <18 months, n (%)	≥18 months and <26 months, n (%)	No exposure, n (%)	*P* value	
**Child’s sex**					.66							.29
	Female	441 (89.82)	50 (10.18)			131 (26.68)		111 (22.61)	40 (8.15)	209 (42.57)		
	Male	503 (89.66)	58 (10.34)			166 (29.59)		139 (24.78)	33 (5.88)	223 (39.75)		
**Child’s age (mo)**					<.001							—^a^
	<12	305 (96.83)	10 (3.17)			—		—	—	—		
	≥12 and <18	328 (93.45)	23 (6.55)			—		—	—	—		
	≥18 and <26	311 (80.57)	75 (19.43)			—		—	—	—		
**Firstborn**					.65							.31
	No	526 (90.22)	57 (9.78)			157 (26.93)		133 (22.81)	46 (7.89)	247 (42.37)		
	Yes	418 (89.13)	51 (10.87)			140 (29.85)		117 (24.95)	27 (5.76)	185 (39.45)		
**Caregiver’s relationship**					.97							.66
	Mother	658 (90.01)	73 (9.99)			210 (28.73)		175 (23.94)	48 (6.57)	298 (40.77)		
	Father	82 (89.13)	10 (10.87)			22 (23.91)		27 (29.35)	6 (6.52)	37 (40.22)		
	Grandparent	197 (89.14)	24 (10.86)			62 (28.05)		45 (20.36)	18 (8.14)	96 (43.44)		
	Others	7 (87.50)	1 (12.50)			3 (37.50)		3 (37.50)	1 (12.50)	1 (12.50)		
**Caregiver’s sex**					.09							.57
	Female	821 (90.42)	87 (9.58)			260 (28.63)		208 (22.91)	63 (6.94)	377 (41.52)		
	Male	115 (85.19)	20 (14.81)			35 (25.93)		38 (28.15)	10 (7.41)	52 (38.52)		
**Caregiver’s age (y)**					.98							.11
	<25	124 (90.51)	13 (9.49)			54 (39.42)		27 (19.71)	9 (6.57)	47 (34.31)		
	≥25 and <30	316 (89.27)	38 (10.73)			89 (25.14)		94 (26.55)	25 (7.06)	146 (41.24)		
	≥30 and <40	272 (90.67)	28 (9.33)			78 (26.00)		76 (25.33)	16 (5.33)	130 (43.33)		
	≥40 and <50	74 (89.16)	9 (10.84)			21 (25.30)		19 (22.89)	5 (6.02)	38 (45.78)		
	≥50	151 (88.82)	19 (11.18)			52 (30.59)		31 (18.24)	17 (10.00)	70 (41.18)		
**Caregiver’s education**					.92							.75
	Junior high school or below	567 (89.43)	67 (10.57)			183 (28.86)		144 (22.71)	43 (6.78)	264 (41.64)		
	High school or above	367 (90.17)	40 (9.83)			110 (27.03)		102 (25.06)	29 (7.13)	166 (40.79)		
**Depression**					.03							.09
	No	742 (90.71)	76 (9.29)			220 (26.89)		192 (23.47)	58 (7.09)	348 (42.54)		
	Yes	191 (87.21)	28 (12.79)			68 (31.05)		55 (25.11)	14 (6.39)	82 (37.44)		
**Anxiety**					.14							.01
	No	700 (90.56)	73 (9.44)			199 (25.74)		181 (23.42)	55 (7.12)	338 (43.73)		
	Yes	229 (88.08)	31 (11.92)			88 (33.85)		64 (24.62)	17 (6.54)	91 (35.00)		
**Stress**					.16							.03
	No	807 (90.37)	86 (9.63)			235 (26.32)		215 (24.08)	64 (7.17)	379 (42.44)		
	Yes	126 (86.90)	19 (13.10)			54 (37.24)		33 (22.76)	8 (5.52)	50 (34.48)		

^a^Not available.

**Table 10:** Differences in sample characteristics between different screen exposure content and coviewing groups.

			Duration of watching educational content (min; n=562)								Frequency of screen exposure with interaction (n=553)					
			<15, n (%)		≥15, n (%)		*P* value			Less than half, n (%)			More than half, n (%)		*P* value	
**Child’s sex**								.05								.39
	Female	213 (84.19)		40 (15.81)					162 (64.03)			91 (35.97)				
	Male	239 (77.35)		70 (22.65)					184 (60.13)			122 (39.87)				
**Child’s age (mo)**								<.001								.20
	<12	93 (90.29)		10 (9.71)					772 (96.02)			32 (3.98)				
	≥12 and <18	155 (84.24)		29 (15.76)					108 (58.70)			76 (41.30)				
	≥18 and <26	204 (74.18)		71 (25.82)					166 (61.25)			105 (38.75)				
**Firstborn**								.37								.07
	No	250 (81.97)		55 (18.03)					198 (65.56)			104 (34.44)				
	Yes	202 (78.60)		55 (21.40)					148 (57.59)			109 (42.41)				
**Caregiver’s relationship**								.09								.68
	Mother	315 (78.16)		88 (21.84)					245 (61.25)			155 (38.75)				
	Father	39 (84.78)		7 (15.22)					25 (56.82)			19 (43.18)				
	Grandparent	94 (87.85)		13 (12.15)					71 (65.74)			37 (34.26)				
	Others	4 (66.67)		2 (33.33)					5 (71.43)			2 (28.57)				
**Caregiver’s sex**								.14								.89
	Female	384 (79.50)		99 (20.50)					300 (62.24)			182 (37.76)				
	Male	64 (87.67)		9 (12.33)					43 (60.56)			28 (39.44)				
**Caregiver’s age (y)**								.08								.46
	<25	61 (75.31)		20 (24.69)					49 (61.25)			31 (38.75)				
	≥25 and <30	148 (76.68)		45 (23.32)					107 (56.61)			82 (43.39)				
	≥30 and <40	129 (81.65)		29 (18.35)					103 (65.61)			54 (34.39)				
	≥40 and <50	37 (92.50)		3 (7.50)					24 (60.00)			16 (40.00)				
	≥50	73 (86.90)		11 (13.10)					58 (67.44)			28 (32.56)				
**Caregiver’s education**								.03								.02
	Junior high school or below	280 (83.83)		54 (16.17)					220 (66.67)			110 (33.33)				
	High school or above	167 (75.91)		53 (24.09)					120 (54.55)			100 (45.45)				
**Depression**								.45								.69
	No	341 (80.42)		83 (19.58)					262 (62.09)			160 (37.91)				
	Yes	103 (81.75)		23 (18.25)					78 (62.40)			47 (37.60)				
**Anxiety**								.36								.35
	No	313 (79.64)		80 (20.36)					236 (60.36)			155 (39.64)				
	Yes	130 (83.33)		26 (16.67)					100 (64.52)			55 (35.48)				
**Stress**								.13								.60
	No	371 (80.30)		91 (19.70)					280 (61.14)			178 (38.86)				
	Yes	76 (83.52)		15 (16.48)					61 (66.30)			31 (33.70)				

## Discussion

### Principal Findings

This study aims to describe the status quo of screen exposure among children aged <3 years in rural China and examine the relationship between the duration, content, and coviewing of screen exposure and ECD. To our knowledge, this is the first study to focus on screen exposure among children aged <3 years in rural China. Our analysis revealed 3 main findings. First, we found that 28.23% (297/1052) of the children were first exposed to screens before the age of 12 months, and the average daily screen time among children who had been exposed was 28 (SD 38.90) minutes. Second, children exposed to screens before the age of 12 months and those with longer screen time between the ages of 12 and 18 months were significantly more likely to experience motor developmental delays than children with later initial exposure or shorter screen time, respectively, but they showed no significant differences in cognitive, language, or socioemotional development. Third, children exposed to educational content for ≥15 minutes on a daily basis had fewer social-emotional or behavioral problems than those with no screen exposure and fewer motor developmental delays than those exposed to educational content for <15 minutes on a daily basis. Caregiver interaction during screen exposure was also associated with fewer cognitive and language developmental delays and better socioemotional skills.

Expanding on our first major finding, we found that the majority (620/1052, 58.94%) of children in the sample had been exposed to screens by the age of 3 years, with 28.23% (297/1052) of them first exposed before 12 months of age. The prevalence of early screen exposure in our sample still exceeds the reasonable range recommended by the World Health Organization, but it is far lower than the level found in high-income societies [29]. The average daily screen time of 27.57 (SD 38.90) minutes is lower than the level previously found in urban areas [30]. Besides geographic environment [10], seasonal trends in screen time [31], digital infrastructure in the area [32], and caregiver’s screen use [33], all of which have been found to affect children’s screen exposure, the relatively low screen exposure in our sample could also be explained by the prevalence of intergenerational caregiving in the region. Because 20% (221/1052) of the children’s primary caregivers in our sample are grandparents, the digital barrier faced by older caregivers may have served as a protective factor and reduced children’s screen exposure.

Our finding that longer screen time among children aged between the ages of 12 and 18 months was only associated with more motor developmental delays is consistent with another study that found no significant association between screen time and behavioral issues [10]. However, other longitudinal and cross-sectional studies have found positive correlations between excessive screen exposure and cognitive, language, and motor delays in children [13,30,34]. This inconsistency can be attributed to the differences in factors that mediate the relationship between screen time and behavioral problems [35]. Screen time may negatively affect development by displacing other beneficial activities, such as exercise, social interactions with friends, and good sleep hygiene [36]. Therefore, increased screen exposure may not lead to developmental issues when the increase is not at the cost of physical activity and sleep. Another possible explanation is the latency of screen time’s effects. Research suggests that the effects of excessive screen exposure in early childhood may not become apparent until after the age of 4 years [37], but it can continue to impact academic performance once children start school [38,39]. Because existing studies mainly focus on screen exposure among children aged <5 years, more longitudinal studies are needed to confirm the long-term effects of screen exposure [35].

Studies suggest that assessing the association between screen exposure duration and ECD in isolation from the content and coviewing of screen exposure fails to capture their effects on the association [40]. Because there is little previous research that examines these 3 dimensions of screen exposure in junction [41], this study aims to assess whether the effect of screen time varies by the content and coviewing of exposure. We found that children exposed to educational content for ≥15 minutes on a daily basis did not experience more developmental delays than children with no screen exposure. In addition, they experienced fewer motor developmental delays than children who spent <15 minutes of their daily screen time on educational content. This suggests that rather than advising against screen time altogether, health care practitioners should encourage parents to intervene in their children’s screen time and select content for them to maximize the developmental benefits of screen exposure.

The associations we found between increased caregiver interaction during screen exposure, fewer cognitive and language delays, and enhanced social-emotional skills are consistent with the findings of Muppalla et al [40], who suggested that caregivers can actively moderate the impact of screen time on children through their involvement [40]. For example, verbal engagement during coviewing maintains a positive caregiver-child interaction, which mitigates the adverse effects of screen media use on early language development [18,42]. If screen time provides opportunities for turn-taking conversations, it can even become a stimulating prompt for parent-child engagement that benefits vocabulary growth [43,44]. However, other research has found that coviewing does not reduce the risk of language development delays when caregivers engage in more narration as opposed to interactive conversations during coviewing [45]. Future studies should explore how different modes of interaction mediate the impact of coviewing on language and socioemotional development.

A novel contribution of this study was the examination of demographic characteristics of households in rural China in which children were less likely to be exposed to high-quality content and interaction during screen time. We found that older children and children whose caregivers exhibited symptoms of depression tended to have longer screen time, while children whose caregivers had symptoms of stress and anxiety were more likely to be introduced to screens at an early age. In addition, children with less educated caregivers experienced less interactive screen exposure. These findings illustrate the interconnected nature of ECD and maternal mental health, and urging practitioners to combine guidance on screen use with efforts to provide mental health support to caregivers. Potential interventions could include using electronic monitoring devices to limit screen time [46], information education about the effects of screen exposure on newborns and young children for mothers during prenatal and postnatal periods [47], and offering targeted advice to new parents during health visits [47].

### Limitations

This study has several limitations. First, we did not measure the relationship between screen time and time spent on sleep, social interaction, and physical activity. Future comprehensive measures of children’s time use will help determine whether screen time impacts development by displacing other activities that support healthy development. Second, this study is a cross-sectional study. Considering the potential latency of screen exposure’s effects on development, we plan to continuously collect data from this study’s sample group and incorporate trend changes observed in our follow-up surveys into future works. Third, this study relied on caregivers’ reports of their children’s screen exposure duration, content, and coviewing. Because we did not directly observe the participants, social desirability bias and recall bias may affect caregivers’ reports of their children’s screen use. In addition, the rapid proliferation of new technologies has changed the way children and their caregivers receive information. Studies have found that on-demand services and video games offer children new forms of entertainment, while time spent watching television has declined [35]. Because active and passive screen time have different mechanisms of action [48], distinguishing between types of screen time in future research will allow a more nuanced understanding of their effects on ECD.

### Conclusions

This study comprehensively considers the duration, content, and coviewing of children’s screen exposure to provide a thorough understanding of the screen exposure habits of children aged <3 years in rural areas of China and its impacts on ECD. A key finding is that the type of content viewed and how caregivers engaged in children’s screen time were more associated with developmental outcomes than the amount of screen time itself. This finding points to the importance of assessing quality in addition to duration in understanding the impact of screen exposure on development. Further research with representative samples from rural areas is needed to explore the relationship between screen time and other developmentally critical activities, such as sleep and physical activity, and to assess its short- and long-term effects on cognitive, language, motor, and social-emotional development, as well as academic performance. Moreover, organizations and policy makers in public health and education need to recognize the limitations of enforcing simple screen time limits for children. Guiding parents to select educational content for their children and engaging in interactive coviewing may better protect children from the negative effects of screen exposure than merely advising against screen time altogether.

## Appendix

Multimedia Appendix 1 Simplified version of the third edition of the Bayley Scales of Infant and Toddler Development assessment, specific statistical formulas and summary statistics of subgroup.

Multimedia Appendix 2 Subgroup analysis on screen exposure duration and child development.

## Abbreviations

Bayley-III: third edition of the Bayley Scales of Infant and Toddler Development
BITSEA: Brief Infant Toddler Social Emotional Assessment
ECD: early childhood development
OLS: ordinary least squares

## Data Availability

The datasets generated or analyzed during this study are available from the corresponding author on reasonable request.
