# Supplementary material for: Screen Exposure and Early Childhood Development in Resource-Limited Regions: Findings From a Population-Based Survey Study
Source: J Med Internet Res. 2025 May 15;27:e68009. doi: 10.2196/68009 (PMC12123236; doi:10.2196/68009)
Supplement: Multimedia Appendix 1 [file jmir_v27i1e68009_app1.docx]

**Appendix**

**Part A: Simplified version of the Bayley-III assessment**

Although widely considered the gold standard for early development evaluation, the iterative nature of Bayley-III makes it more time-consuming and expensive than other assessment methods. To optimizing the cost-effectiveness of the measure while preserving its informational value and the distribution of difficulty levels, we followed the detailed methodology outlined by Emmers et al. (2024) and constructed a simplified version of Bayley-III by selecting 15 items from each scale.

Children were stratified by gender and age before being randomly assigned to receive either the complete or simplified version of the Bayley-III assessment. Assessors who participated in a one-week practice training prior to data collection administered the assessment using a standardized set of toy kits and detailed record sheets. The simplified assessment showed a Kuder-Richardson coefficient of above 0.70, indicating its reliability, and a Pearson correlation coefficient of above 0.90 to scores from the full Bayley-III, indicating its validity. We used binary scores from the Bayley-III items to construct aggregated, continuous cognitive skills, language, and motor skills scores. We estimated a one-parameter logistic item response theory (1PL IRT) model to construct ECD factor scores.

**A.1 ECD outcomes**

ECD outcomes are measured using the Bayley-III cognition, language, and motor scales for half of the sample, while we administered 15-item short forms containing a subset of the Bayley-III items to the other half of the sample (see Emmers et al. (2024) for a detailed description of the item selection and validation procedure). Stratified by age (in months) and sex, we randomly assigned study subjects to Bayley-III benchmark or ECD short form assessment. For each of the cognition, language, and motor scales, administered test items are increasing in difficulty level. Given this specific test structure, we estimate a one-parameter logistic item response theory (1PL IRT) measurement system for each of the ECD scales, which calculates the optimal weighted average of all items taking into account differences in difficulty levels of test items. To be specific, we fit the following logistic 1PL IRT model to the unidimensional underlying skill factor:

$$m_{j,k,t}=\mu_{j,k,t}+\alpha_{k,t}\vartheta_{k,t}+\varepsilon_{j,k,t} (A.1)$$

Where $m_{j,k,t}$ is the observable response to test item *j* of factor *k* at time *t*, $\mu_{j,k,t}$ is the difficulty level of item *j*, $\vartheta_{k,t}$ is the underlying latent skill factor *k* at time *t*, and $\alpha_{k,t}$ is the factor loading of factor *k* at time *t* measuring the average discriminatory power of the test items (An & Yung, 2017). A higher discrimination parameter means that the probability of completing the test item differs substantially between two children with only slightly different latent skill levels, thus allowing us to distinguish between minor differences in skills. Higher discriminatory power indicates that the test item contains more information on the underlying skill factor (Edelen & Reeve, 2007). The estimation of the described 1PL IRT model relies on the assumption that no more than one latent skill factor $\vartheta_{k,t}$ underlies the responses to the test items of the Bayley cognition and motor scales.

Note that latent factors have no natural metric. Therefore, we need to impose some normalizing restrictions to set the location and scale across the different versions of the Bayley test (Anderson & Rubin, 1956). Expressing the scale of the latent factor arbitrarily, by normalizing the loading on a different item in the Bayley-III benchmark and the short form assessments, would imply that the technology parameters themselves would be expressed in different metrics across the samples that used different assessments. However, a straightforward solution is readily available if we make use of the overlapping test items between the different versions of the test. We can easily express the location and scale of the factor in terms of these overlapping test items by imposing that the difficulty and discriminatory power of overlapping items is fixed across different versions of the test.

Appendix Figure A.1 plots the distribution of the estimated item-level difficulty parameters, $\mu_{j,k,t}$, for the Bayley-III and short form cognition items from the logistic 1PL IRT measurement system. Items with negative difficulty parameters are easier, because children with a comparatively low latent skill level are likely to be able to accomplish this test item. Items with positive difficulty parameters are comparatively harder, because a higher latent skill factor is required for children to be able to pass this test item. The distribution of the estimated difficulty parameters provides information about whether the test is well-designed for the population under study. In an ideal test, the difficulty parameters smoothly transition from easy to more difficult and cover the whole skill distribution. By this metric, the tests are fairly well-designed, because the test items are able to distinguish reasonably well between children with low, medium, and high skill levels.

**Appendix Figure A.1**

*Difficulty of Cognition Items*


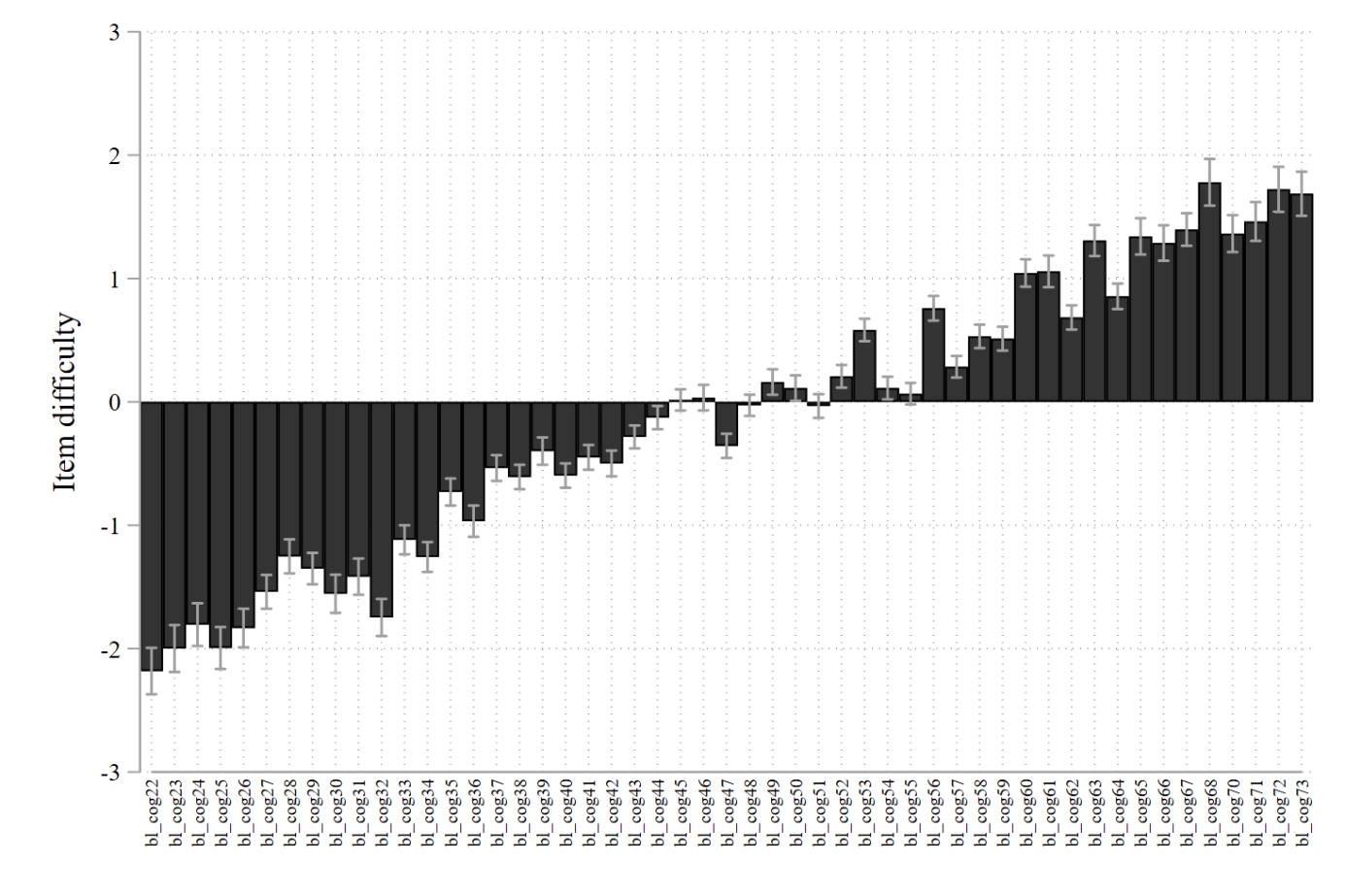


Appendix Figures A.2 and A.3 plot the distribution of the estimated difficulty parameters, $\mu_{j,k,t}$, for the language and motor items, respectively. The language and motor items are suitable to distinguish between moderately low and high skill levels. However, they are not useful to distinguish skill levels at the top and bottom tails of the distribution.

**Appendix Figure A.2**

*Difficulty of Language Items*


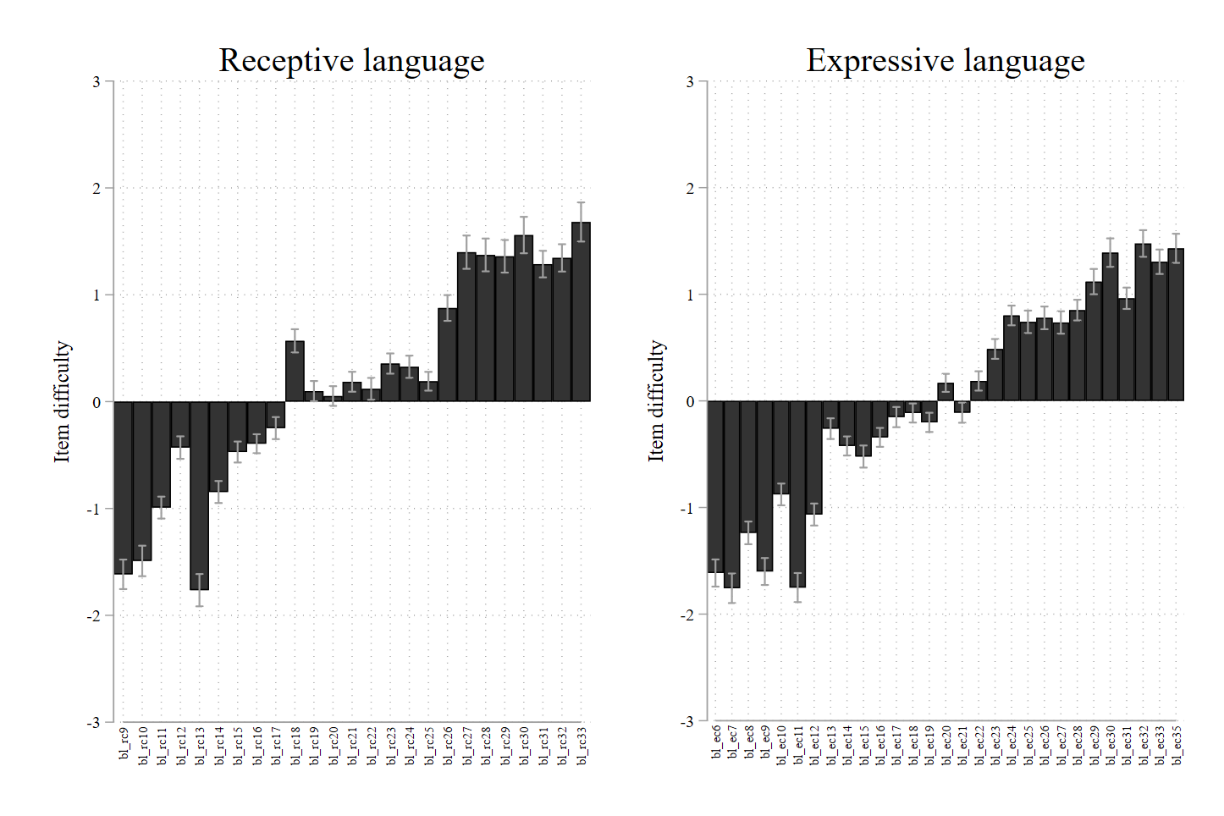


**Appendix Figure A.3**

*Difficulty of Motor Items*


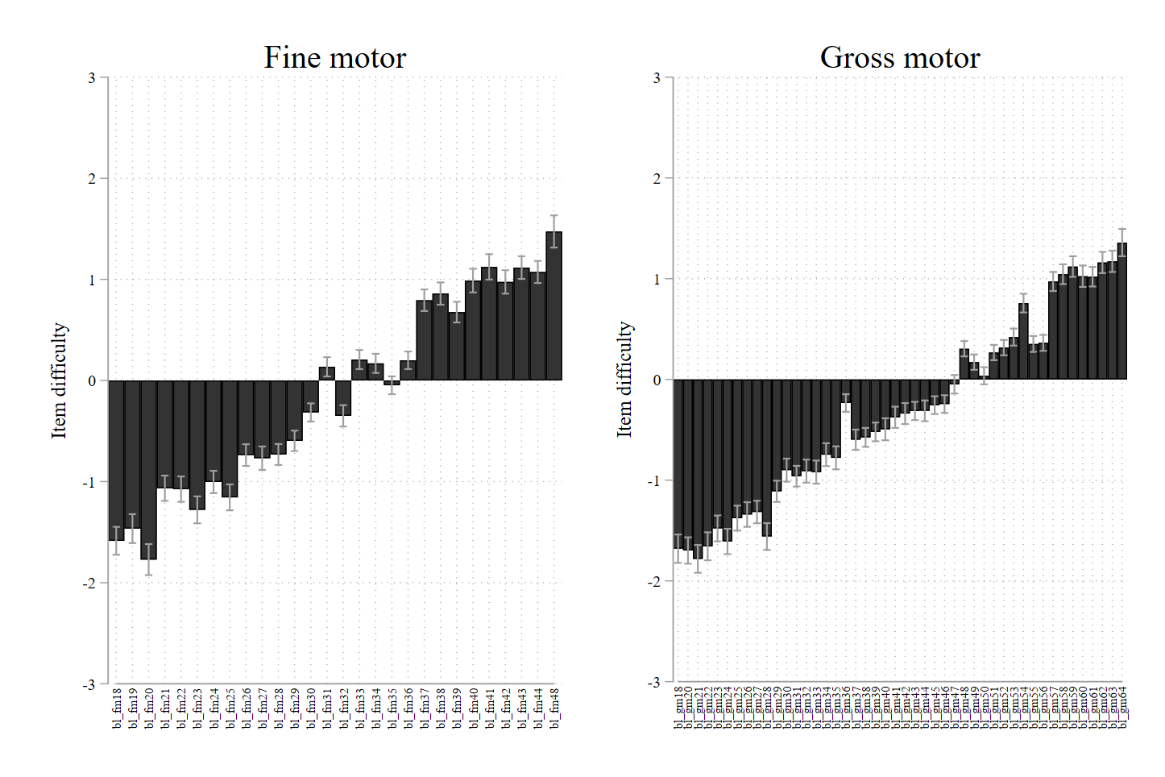


In order to evaluate overall test performance, we also estimate test information functions (TIFs). TIFs measure the amount of information provided by a test at different levels of the underlying trait or ability being measured. As shown in Figure A.4, overall test information is high. The informational value is even higher for the Bayley-III test than for the short forms. Hence both measures are useful skill assessments. The Bayley-III benchmark and short form assessments both provide less information at the tails of the skill distribution.

**Appendix Figure A.4**

*TIFs of Bayley-III and short-form scores*


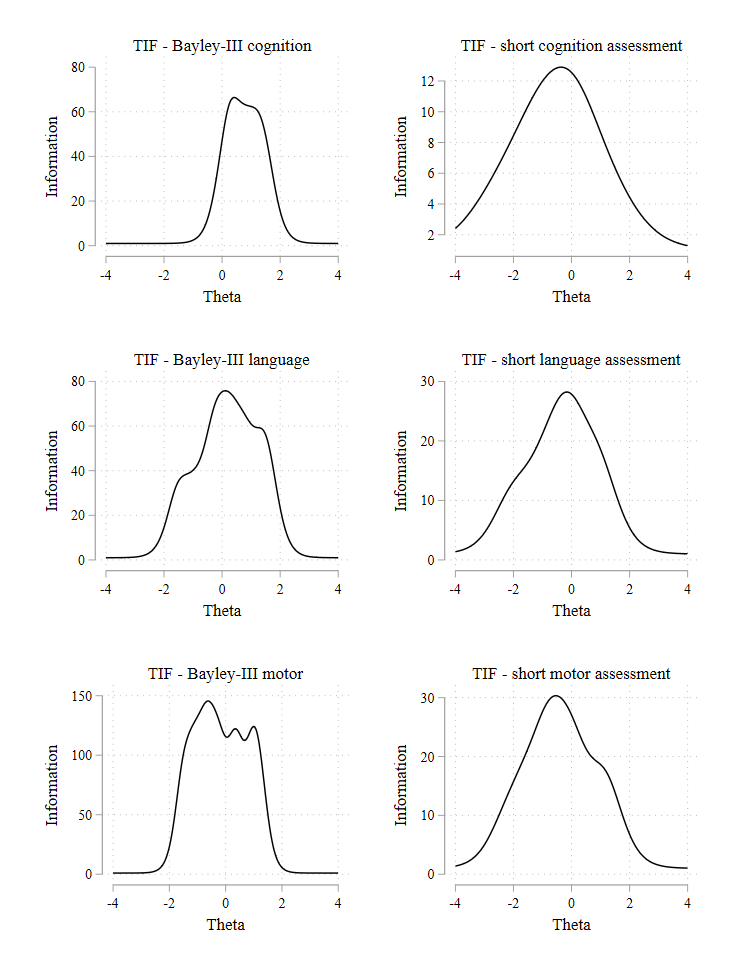


Finally, the cognition, language, and motor factor scores resulting from IRT factor estimation are increasing in age, because skills accumulate and mature rapidly during the first years of life. In order to eliminate this age effect, we use a non-parametric approach to standardize the cognition and motor factor scores withing age (in months) group.

**References**

An, X., & Yung, Y.-F. (2017). Item Response Theory: What It Is and How You Can Use the IRT Procedure to Apply It. *SAS Institute Inc.*

Anderson, T. W., & Rubin, H. (1956). Statistical Inference in Factor Analysis. In *Proceedings of the 3rd Berkeley Symposium on Mathematical Statistics and Probability* (1–5, pp. 111–150).

Cattell, R. B. (1961). Theory of situational, instrument, second order, and refraction factors in personality structure research. *Psychological Bulletin*, *58*(2), 160–174. https://doi.org/10.1037/h0045221

Edelen, M. O., & Reeve, B. B. (2007). Applying item response theory (IRT) modeling to questionnaire development, evaluation, and refinement. *Quality of Life Research*, *16*, 5–18.

Emmers, D., Warrinnier, N., Qian, Y., Wang, L., Zhang, S., Chen, A., Ha, V., Li, R., Zhang, M., Zhu, S., Rozelle, S., & Sylvia, S. (2024). *Big Data on Little People: Toward Psychological Assessment at Scale in Resource-poor Field Settings* [SCCEI Working Paper].

Horn, J. L. (1965). A rationale and test for the number of factors in factor analysis. *Psychometrika*, *30*(2), 179–185. https://doi.org/10.1007/BF02289447

**Part B**

We tested the relationship between the duration of screen exposure and ECD outcomes in different age groups. We estimated the following ordinary least squares (OLS) regression and logistic regression specifications:

*L_i_=α+β_1_F_i_+β_2_C_i_+ε_i_*  (1)

*L_i_=α+β_1_Q_i_+β_2_C_i_+ε_i_*  (2)

$Yi=\frac{e^{b1+b2Fi+b3Ci}}{1+e^{b1+b2Fi+b3Ci}}$ (3)

$Yi=\frac{e^{b1+b2Qi+b3Ci}}{1+e^{b1+b2Qi+b3Ci}}$ (4)

Where *Y_i_* is a binary variable of cognition, language, or motor skills of child *i*; *L_i_* is standardized z-score for competence delays and social-emotional problems of child *i*; *F_i_* is the age of first screen exposure of child *i*; *Q_i_* is the average time (in minutes) of screen exposure of child *i* in each day in the past month; *C_i_* is a list of covariates for child *i* that includes their age, gender, whether they were firstborn, household asset index, household electronic devices index, caregiver gender, caregiver age, caregiver education level, and caregiver’s relationship to the child. Standard errors are clustered at the individual level.

Similarly, we tested whether the content and context of screen exposure impacted ECD outcomes by estimating the following OLS regression and logistic regression specifications:

$L_{i}=\alpha+\beta_{1}{Qc}_{i}+\beta_{2}C_{i}+\varepsilon_{i}$ (5)

$L_{i}=\alpha+\beta_{1}{Ql}_{i}+\beta_{2}C_{i}+\varepsilon_{i}$ (6)

$L_{i}=\alpha+\beta_{1}{{Xa}_{i}+\beta}_{2}C_{i}+\varepsilon_{i}$ (7)

$L_{i}=\alpha+\beta_{1}{{Xe}_{i}+\beta}_{2}C_{i}+\varepsilon_{i}$ (8)

$Yi=\frac{e^{b1+b2{Qc}_{i}+b3Ci}}{1+e^{b1+b2{Qc}_{i}+b3Ci}}$ (9)

$Yi=\frac{e^{b1+b2{Ql}_{i}+b3Ci}}{1+e^{b1+b2{Ql}_{i}+b3Ci}}$ (10)

$Yi=\frac{e^{b1+b2{Xa}_{i}+b3Ci}}{1+e^{b1+b2{Xa}_{i}+b3Ci}}$ (11)

$Yi=\frac{e^{b1+b2{Xe}_{i}+b3Ci}}{1+e^{b1+b2{Xe}_{i}+b3Ci}}$ (12)

Where *Y_i_* is the cognition, language, or motor binary variable of child *i*; *L_i_* is the competence delays and social-emotional problems standardized z-score of child *i*; ${Qc}_{i}$ is a categorical variable denoting whether child *i* spends more time watching child-friendly or non-child-friendly content daily (child-friendly versus non-child-friendly versus no exposure); ${Ql}_{i}$ denotes the average time child *i* spends daily watching educational content (<15 minutes versus ≥15 minutes versus no exposure); ${Xa}_{i}$ denotes whether child *i* spends more than half of their daily screen exposure in the company of others (less than half versus more than half versus no exposure); ${Xe}_{i}$ denotes whether child *i* spends more than half of their daily screen exposure interacting with a family member (less than half versus more than half versus no exposure); *C_i_* is a list of covariates for child *i* that includes their age (<12 months/ ≥ 12 & < 18 months /≥ 18 & < 26 months), gender (female/male), whether they were firstborn (yes/no), household asset index (range: 0-1), caregiver gender (female/male), caregiver age (<25 years/ ≥25 & <30 years /≥30 & <40 years /≥40 & <50 years /≥50 years) , caregiver education level (junior high school or below/ high school or above), and caregiver’s relationship to the child (mother/father/grandparent/others). Standard errors are clustered at the individual level.

**Part C**

**Table S1 Characteristics of babies and their primary caregiver by different groups**

| **Variables** |  | **Group A**  **(N=818)** | | **Group B (N=937)** | | **Group C**  **(N=960)** | | **Total**  **(N=1052)** | |
| --- | --- | --- | --- | --- | --- | --- | --- | --- | --- |
|  |  | N | % | N | % | N | % | N | % |
| **Baby’s Gender** |  |  |  |  |  |  |  |  |  |
|  | Female | 382 | 46.70 | 434 | 46.32 | 451 | 46.98 | 491 | 46.67 |
|  | Male | 436 | 53.30 | 503 | 53.68 | 509 | 53.02 | 561 | 53.33 |
| **Baby’ Age Group** |  |  |  |  |  |  |  |  |  |
|  | <12month | 250 | 30.56 | 275 | 29.35 | 273 | 28.44 | 315 | 29.94 |
|  | >=12&<18month | 278 | 33.99 | 309 | 32.98 | 320 | 33.33 | 351 | 33.37 |
|  | >=18&<26month | 290 | 35.45 | 353 | 37.67 | 367 | 38.23 | 386 | 36.69 |
| **Firstborn** |  |  |  |  |  |  |  |  |  |
|  | No | 458 | 55.99 | 519 | 55.39 | 527 | 54.90 | 583 | 55.42 |
|  | Yes | 360 | 44.01 | 418 | 44.61 | 433 | 45.10 | 469 | 44.58 |
| **Type of Caregiver** |  |  |  |  |  |  |  |  |  |
|  | Mother | 570 | 69.68 | 655 | 69.90 | 672 | 70.00 | 731 | 69.49 |
|  | Father | 70 | 8.56 | 85 | 9.07 | 86 | 8.96 | 92 | 8.75 |
|  | Grandparent | 172 | 21.03 | 190 | 20.28 | 195 | 20.31 | 221 | 21.01 |
|  | Others | 6 | 0.73 | 7 | 0.75 | 7 | 0.73 | 8 | 0.76 |
| **Caregiver’s Gender** |  |  |  |  |  |  |  |  |  |
|  | Female | 712 | 87.90 | 806 | 86.76 | 828 | 86.88 | 908 | 87.06 |
|  | Male | 98 | 12.10 | 123 | 13.24 | 125 | 13.12 | 135 | 12.94 |
| **Caregiver’s Age Group** |  |  |  |  |  |  |  |  |  |
|  | <25 | 100 | 12.22 | 126 | 13.45 | 132 | 13.75 | 137 | 13.02 |
|  | ≥25&<30 | 273 | 33.37 | 321 | 34.26 | 325 | 33.85 | 354 | 33.65 |
|  | ≥30&<40 | 239 | 29.22 | 265 | 28.28 | 272 | 28.33 | 300 | 28.52 |
|  | ≥40&<50 | 64 | 7.82 | 71 | 7.58 | 77 | 8.02 | 83 | 7.89 |
|  | ≥50 | 136 | 16.63 | 147 | 15.69 | 147 | 15.31 | 170 | 16.16 |
| **Caregiver’s Education** |  |  |  |  |  |  |  |  |  |
|  | Junior High School and Below | 498 | 60.88 | 562 | 59.98 | 571 | 59.48 | 634 | 60.27 |
|  | High School and Above | 312 | 38.14 | 366 | 39.06 | 379 | 39.48 | 407 | 38.69 |

*Group A is all of the samples who have finished Bayley-III assessment. Group B is all of the samples who have finished Bitsea- social-emotional problems assessment. Group C is all of the samples who have finished Bitsea- competence delays assessment.

**Table S2 Summary Statistics of Baby’s Screen Exposure Quantity by different groups**

| **Variables** |  | **Group A (N=818)** | | **Group B (N=937)** | | **Group C**  **(N=960)** | | **Total**  **(N=1052)** | |
| --- | --- | --- | --- | --- | --- | --- | --- | --- | --- |
|  |  | N | % | N | % | N | % | N | % |
| **Age of First Screen Exposure** |  |  |  |  |  |  |  |  |  |
|  | No Exposure | 347 | 42.42 | 380 | 40.55 | 387 | 40.31 | 432 | 41.06 |
|  | ≥18&<26 Month | 53 | 6.48 | 67 | 7.15 | 72 | 7.50 | 73 | 6.94 |
|  | ≥12&<18 Month | 188 | 22.98 | 228 | 24.33 | 236 | 24.58 | 250 | 23.76 |
|  | <12 Month | 230 | 28.12 | 262 | 27.96 | 265 | 27.60 | 297 | 28.23 |
| **Screen Exposure** |  |  |  |  |  |  |  |  |  |
|  | 0 Hour | 386 | 47.19 | 424 | 45.25 | 426 | 44.37 | 483 | 45.91 |
|  | <1Hour | 344 | 42.05 | 412 | 43.97 | 430 | 44.79 | 461 | 43.82 |
|  | ≥1Hour | 88 | 10.76 | 101 | 10.78 | 104 | 10.83 | 108 | 10.27 |
| **Minutes of Screen Exposure** | Mean, (SD) | 16.63(34.31) | | 16.98(33.90) | | 16.98(33.25) | | 16.25(32.79) | |

*Group A is all of the samples who have finished Bayley-III assessment. Group B is all of the samples who have finished Bitsea- social-emotional problems assessment. Group C is all of the samples who have finished Bitsea- competence delays assessment.
