# Supplementary material for: Screen Exposure and Early Childhood Development in Resource-Limited Regions: Findings From a Population-Based Survey Study
Source: J Med Internet Res. 2025 May 15;27:e68009. doi: 10.2196/68009 (PMC12123236; doi:10.2196/68009)
Supplement: Multimedia Appendix 2 [file jmir_v27i1e68009_app2.docx]

**Table S1.** Screen exposure duration and child development (subgroup age ≥12 and <18 mo).

|  | Age of first screen exposure (≥12 and 18 mo), odds ratio^a^ (95% CI) | | Age of first screen exposure (<12 mo), odds ratio^a^ (95% CI) | | Daily screen time, odds ratio^a^ (95% CI) | |
| --- | --- | --- | --- | --- | --- | --- |
| Delay in cognition (n=278) | 1.698 (0.803-3.589) | | 1.131 (0.521-2.454) | | 0.991 (0.978-1.003) | |
| Delay in language (n=278) | 1.228 (0.578-2.610) | | 1.633 (0.766-3.484) | | 0.997 (0.987-1.009) | |
| Delay in motor (n=278) | 1.042 (0.343-3.165) | | 1.670 (0.588-4.739) | | 1.017^b^ (1.004-1.031) | |
|  | Estimate^a^ | SE | Estimate^a^ | SE | Estimate^a^ | SE |
| Social-emotional behavior problems (n=309) | 0.798 | 0.852 | 0.951 | 0.839 | –0.005 | 0.013 |
| Social-emotional behavior competencies (n=320) | 0.190 | 0.409 | –0.208 | 0.417 | –0.001 | 0.007 |

^a^No exposure group used as reference. Control variables included baby’s age, baby’s gender, firstborn or not, household asset index, type of caregiver, caregiver’s gender, caregiver’s age, and caregiver’s education.

^b^*P*<.05.

^***^*P*<.001.

^**^*P*<.01.

**Table S2.** Screen exposure duration and child development (subgroup age ≥18 and <26 mo).

|  | Age of first screen exposure (≥18 and <26 mo), odds ratio^a^ (95% CI) | | Age of first screen exposure (≥12 and <18 mo), odds ratio^a^ (95% CI) | | Age of first screen exposure (<12 mo), odds ratio^a^ (95% CI) | | Daily screen time, odds ratio^a^ (95% CI) | |
| --- | --- | --- | --- | --- | --- | --- | --- | --- |
| Delay in cognition (n=290) | 0.874 (0.333-2.295) | | 0.827 (0.382-1.794) | | 0.922 (0.387-2.195) | | 1.002 (0.995-1.009) | |
| Delay in language (n=290) | 0.506 (0.177-1.448) | | 1.189 (0.538-2.629) | | 1.027 (0.417-2.527) | | 1.007 (0.996-1.015) | |
| Delay in motor (n=290) | 0.658 (0.170-2.549) | | 0.484 (0.156-1.496) | | 0.396 (0.100-1.568) | | 0.999 (0.988-1.012) | |
|  | Estimate^a^ | SE | Estimate^a^ | SE | Estimate^a^ | SE | Estimate^a^ | SE |
| Social-emotional behavior problems (n=353) | –1.950 | 1.011 | –0.532 | 0.855 | 0.568 | 0.989 | –0.001 | 0.008 |
| Social-emotional behavior competencies (n=367) | 0.194 | 0.486 | 0.203 | 0.416 | 0.875 | 0.474 | 0.001 | 0.004 |

^a^No exposure group used as reference. Control variables included baby’s age, baby’s gender, firstborn or not, household asset index, type of caregiver, caregiver’s gender, caregiver’s age, and caregiver’s education.

^***^*P*<.001.

^**^*P*<.01.

^*^*P*<.05.
